# Filling over Exposed Pulps

**Published:** 1872-10

**Authors:** S. P. Cutler


### ARTICLE II.

### *Filling over Exposed Pulps.*

BY S. P. CUTLER, M.D., D.D.S.

Read before the Tennessee Dental Association.

*Ossified Pulps.*—A young lady, about 20 years, had several decayed teeth; one superior second molar, with large central decay extending deep into the body of the tooth. I excavated sufficiently deep to have reached the pulp in ordinary teeth. At the bottom there was an ossified pulp, of a brownish color, granular in appearance, but firm; not much sensibility of tooth, every way vital. I did not remove any of the new formation; used creasote, then dried and filled with gold; do not anticipate any trouble; it had never given any trouble. Lymphatic nervous temperament.

*Continued Case.*—This same young lady had an opposite upper molar likewise ossified, which I filled in a similar manner. She also had an upper bicuspid, with an extensive approximal decay similarly ossified, which I also filled without any inconvenience, and I have no apprehension of trouble in the future.

*Exposed Pulps.—Case* 1.—A gentleman, 30 years old, had an upper bicuspid aching from exposure of pulp. I removed only the loose and easily detached portions of decay which was very extensive, and filled with os-artificiel; the pain soon ceasing. Creasote was also used before filling; have not heard from the case since.

*Case* 2.—A lady, 30 years old, stout and well formed, has the two upper bicuspids on the left side extensively decayed between, and pulp involved in the decay; had been aching for some time, aching at the time, I partially excavated and filled with os-artificiel; all pain soon ceased; application of warmth entirely relieved all pain; creasote was first applied.

*Case* 3.—A young lady, about 22 years old, had the six year old molars on each side, above, decayed across grinding surfaces, and the enamel all gone on one of them, but the tooth had become smooth from mastication with no cavity in it; the other one was in a similar condition; only in the centre there was a large, shallow cavity, also worn smooth, with some slight sensibility only on excavating one point at the bottom. Both of these teeth were dark from stain of dentine; I filled the tooth with amalgam.

In the same mouth one of the lower wisdom teeth was extensively decayed on the buccal suface—one-third of tooth gone—which I found sensitive at one point, but no exposure of pulp. I capped with a bit of spunk and filled with amalgam. The sensitive point at first pressure caused slight pain, which soon subsided. All these teeth had ossified inward with usual characteristics. Young lady healthy; never having had toothache; all the balance of her teeth good.

*Case* 4.—*Partial Ossification of Pulp, and Tooth Ache.*—A young man, 24 years old, dental student, had an upper right second bicuspid, with a tolerable large post approximal, and a small gold filling on anterior approximal surface. Both cavities had been filled and no trouble was experienced for a long time, but recently commenced aching, and in consequence the large filling had been removed, which gave some relief. I examined the tooth and advised a temporary filling with os-artificiel, as I found only slight sensibility, and no actual exposure or opening. He had it filled and retained it in several hours, all the time aching heavily; he had this filling removed which gave some relief. He now decided to have it removed, which gave much pain, the fang being covered with blood.

On splitting open the tooth, I found the pulp canal very much narrowed in crown portion, with very dark dentine in bottom of both cavities, extending to near nerve pulp, though no actual opening from decay. The pulp was not much inflamed and apparently healthy; no pus or bad scent. He had taken cold in the tooth and jaw. This tooth with a little patience might have been saved. Ossification had gone on but had probably ceased. A great many teeth are lost from want of patience on the part of owners, and often that of the dentist. In fact we dentists, especially when located in towns, might, and I think ought to, study and practice on all such cases, but we are too much afraid of failures and responsibilities, and of other dentists who may take advantage and talk of our failures and injure our practice.

*Case* 5.—A gentleman, about 40 years of age, has but the two canines in his upper jaw, all the balance having been lost long since. He has been wearing a gold plate for a long time attached to these two teeth, the left one having a gold filling and two decays, one on either side, extending to gums, one being very extensive and deep, exposing the original site of the pulp canal, which has been undergoing secondary changes for a considerable time; the tooth being partially loosened from the plate clasps, which loosened condition, no doubt, has had some influence on the ossifying process.

The cavity was slightly sensitive in the bottom, and extended nearly half through the tooth. The pulp did not present the whitish appearance which is so often met with; on the contrary, a more normal appearance, or more fleshy, though hard and firm. I filled both sides of the tooth; do not anticipate any trouble whatever.

*Case* 6.—A young lady 20 years old, had right upper bicuspid filled on posterior approximal surface with a welded gold filling, several years since. I found the filling loose, ready to drop out I excavated the cavity which extended over the whole side, one-third the thickness of tooth in depth, and no exposure of nerve, some sensibility; color normal and vital at the bottom of cavity, the pulp having ossified down under the exposure. I filled with gold; some sensibility to cold water; I apprehend no further trouble; she has a number of others filled; she is hearty.

*Case* 7.—A gentleman about 40 years old had a large filling inserted into the right superior first molar, in the grinding surface about 18 years ago, which had recently come out. The tooth had never given him any trouble; on examination, at two points of what was originally the points of almost exposure of pulp, was distinctly visible secondary dentine of a different color from the other; sound and firm, but slightly sensitive to the excavations, but not as sensitive at the portions up near the enamel. I refilled with gold, with all confidence of entire success.

*Case* 8.—A man about 30 years old, has bad teeth, and a number missing. The superior molar and right side crown mostly gone; had ached about ten years ago, since had not ached until yesterday, when it became intolerable and lasted all night; I extracted it this morning, January 27th, 1872. On examination I found the original pulp cavity exposed, but the pulp intact and partially ossified, and one point exposed; the main cavity brown, and in a chronic condition; not decaying though it had not been used in ten years, owing to non-antagonism. The fangs came out white and clean, and no inflammation at all; the pulp in one fang mostly ossified, portion in the other fangs not ossified; some inflammation in remaining portions of pulp; the point of exposure no doubt causing the trouble.

*Case* 9.—A married lady 30 years of age, has a number of decayed teeth; all her teeth very sensitive from nervous temperament. The left superior wisdom tooth has a large decay on posterior side, white decay and very sensitive; also central superior incisor. I filled with os-artificiel temporarily on January 15th, 1872. On February 27th, removed temporary fillings, and found sensibility mainly gone in both; dentine firm and sound; pulp in the wisdom tooth must have been exposed at the time of temporary stopping, as it stood out like a pearl slightly above floor of cavity clear, white, and solid, and not sensitive only near enamel, at one point. It has given no pain at all since I first filled it. My opinion is, that this ossification has taken place since January 15th; there may have been a saturation of lime salts in the pulp at the time of first operating, though this is hardly probable, from the fact that there is always an acid condition in all such decays, which theoretically would prevent ossification; I filled with amalgam and apprehend no further trouble.

*Case* 10.—A man about 30 years of age, good health, nervous bilious habit, has a left lower molar decayed on grinding surface, down into the pulp chamber, which is filled with hard firm dark-brown secondary dentine, slightly sensitive, the brown appearance extending into the normal dentine of crown on sides of cavity near the bottom. In all respects the tooth is perfectly healthy and vital, not having caused any trouble. I filled with gold, and no trouble is apprehended whatever: there are a few other teeth only decayed and one or two missing.

*Case* 11.—A gentleman 35 years of age, good constitution; right lower wisdom tooth the crown mostly decayed away, the anterior wall standing, and a small portion of posterior "wall." I found on removal of decay, a sound and healthy floor lined with gum, the pulp beautifully ossified and white, only sensibility to water and excavator. I filled with amalgam, and anticipate no trouble whatever, as it had never given any.

*Case* 12.—A gentleman 58 years old, good sound condition, has a splended set of teeth, only one missing. A central prox. incisor cavity was filled 40 years ago; is sound and perfect as any filling I ever saw, takes unusual care of his teeth, so much so, that he has cross grooved with the brush the left lower cuspid in several places through the enamel and the centrals to some extent, also the sup. right cuspid deeply grooved, and the centrals scratched; uses barks and cream of tarter as powder.

The first right lower molar has two large cavities in anterior and posterior prox. surfaces, the anterior one very large, extending to pulp cavity, which a dentist had attempted to pull, and failing had filled with amalgam, which remained in only a few weeks. The pulp is beautifully ossified, showing distinctly the full amount of exposure, of a light hyaline appearance, the posterior cavity sensitive, and ached when food was crowded down in mastication. On excavating I found some sensibility and evidently the nerve much nearer than in the larger decay, or not so much ossified, I filled both with amalgam, and have no apprehensions of results; he came in thinking the tooth would have to be lost.

*Case* 13.—A lady with all of her upper teeth out except two molars on one side and one on the other. The second molar had been filled with amalgam on the side, during the war, a very large cavity, and was in still, but decayed around it so as to require refilling. On removing the filling I found the pulp completely ossified and very hard and no sensibility, still vital and life-like in all respects. She has very sensitive teeth wherever decayed; nervo-sanguine temperament; I refilled it with amalgam and anticipated no difficulty. This pulp must have been slightly exposed at the time it was fresh filled, and the dark oxide of silver healed the pulp and prevented death of the same; gold would have caused its death.

*Case* 14.—A healthy lady, some 28 years old, has fine teeth generally, has the sup. lateral incisor on right proximal surface decayed, quite a cross from apex to cervex; had been decayed 15 years; the decay is of the dark chronic variety, solid and firm dentine, with the pulp firmly ossified, is of a brown color, not sensitive; I filled with soft foil and have no apprehension of results.

All such cases make interesting specimens for microscopic observation. Any one at all conversant with microscopy or normal or primary dentine, will at once recognize the striking difference between the primary and secondary specimens, and will be able to trace out the tubular continuation between the two, proving conclusively the nerve fibrillar structure of the tubuli, i. e. that each contains a nerve fibril, otherwise when this inward ossification took place, there would be no continuation of tubes inward, the tissue would all be ossified alike, the nerve fibrils being so highly vitalised, that they resist the ossific encroachments, hence, the process takes place in the surrounding cellular and connective tissue structures, leaving the nerve fibrils uninterupted, and the tubular structure referred to is formed during the process of hardening.

The lime deposits at first are a soft structure, but hardens by absorption of the more watery elements, leaving a semi-crystalline structure among the nerve fibrils. This process does not differ materially from that of petrifactions out of the body, being a downward process; dominating vitality in the part where enacted—otherwise the process could not happen. Although a mineralising process, it is in many cases a conservative and protective one, fulfilling normal purposes in "tooth economy."

Without the aid of the microscope, this process of secondary ossification would be recognized in practice, and a more or less practical knowledge attained; though like the natural structures of teeth, very little accurate knowledge of what this structure was, would be possessed by the profession, which actually was the case but a very few years back. The microscope is the revelator of all true organic structures; without its penetrating powers into microcosmic invisibility to "unaided vision," we now would be but little more advanced than our ancestral "tonsorial barbarismic" professional progenitors of past centuryal fame.

They were the pioneers; they had to be before we could be; they had no microscopes; they had not then been invented; hence all tooth anatomy then known was simply descriptive,not histological and descriptive combined,as now understood and taught in our text books.

The microscope makes us familiar with structures so small, that their very existence would not be guessed at even without its aid; these microscopic structures are the true organic structures; they are the organic units that in their aggregations form organs and animals. From my recent discoveries, I am now fully convinced that there is nothing so small that 500 diameters will not reach and reveal, even the ultimate atoms of all known or unknown matter. This statement at this time may be somewhat paradoxical, and may require time before it receives full recognition by scientific men; it is however only a question of time, like all other new and startling truths, that have heretofore been promulgated.

